# Editorial: Aquaporins: Dynamic Role and Regulation

**DOI:** 10.3389/fpls.2017.01420

**Published:** 2017-08-15

**Authors:** Rupesh K. Deshmukh, Henry T. Nguyen, Richard R. Belanger

**Affiliations:** ^1^Département de Phytologie–Faculté des Sciences de l'Agriculture et de l'Alimentation, Université Laval Québec, QC, Canada; ^2^National Center for Soybean Biotechnology and Division of Plant Sciences, University of Missouri Columbia, MO, United States

**Keywords:** water transport, solute specificity, expression profiling, genome wide analysis, protein characterization

Aquaporins (AQPs), a class of channel forming integral membrane proteins, play a vital role in the transport of water and many other small solutes in most living organisms. In higher groups, AQPs have evolved in many isoforms with diverse solute specificity, subcellular localization, expression profile, interactions, and regulation mechanisms. Plant AQPs are mainly classified into five subfamilies based on phylogenetic relationships: plasma membrane intrinsic proteins (PIPs), nodulin 26-like intrinsic proteins (NIPs), tonoplast intrinsic proteins (TIPs), small intrinsic proteins (SIPs), and uncharacterized intrinsic proteins (XIPs) (Quigley et al., 2001; Deshmukh et al., 2015). PIPs and TIPs are predominantly involved in water transport, and represent a class of highly expressed AQPs in root and vegetative tissues. For their part, NIPs represents a relatively diverse group of AQPs with very specific expression profiles. They are known to be involved in the transport of metalloids such as silicon (Si), boric acid, germanium, and arsenic. The two remaining subfamilies SIP and XIP consist of only a few members, with the XIPs being present in only a few dicots.

The AQP transport system is highly regulated and complex. Aquaporin channels harbor a precise level of solute specificity and have evolved with a gating mechanism through which the channels can be turned on and off. Aquaporins are known to interact with each other and with other proteins, leading to a regulation of several physiological processes in the plant. For instance, AQPs are involved in root and shoot elongation, source to sink movement of nutrients, seed development, and other specific physiological processes such as stomatal movement, pollen hydration and germination (McGaughey et al.; Pérez Di Giorgio et al.; Song et al.). In recent years, owing to their role in water transport activity and other physiological functions, AQPs have become of great interest for crop improvement programs aimed at developing stress tolerant varieties. As a result, a better understanding of the complex transport system under AQP regulation is required to optimize efficient movement of water and other solutes necessary for healthy plant development, particularly under stress conditions.

## Solute specificity and role of conserved features in aquaporins

Solute specificity of AQPs is known to be governed by many conserved features such as NPA motifs and aromatic/arginine (ar/R) selectivity filter (SF). Unlike NPA motifs, which are conserved across all AQPs, the amino acid (AA) sequences at the ar/R SF are more specific to subfamilies, or even groups within a subfamily. In addition, the functional impact of several other conserved AA, or even the AA distance between conserved features have been highlighted with mutagenesis studies (Deshmukh et al., 2015; Kirscht et al.). The recent study by Kirscht et al. represents a classic case where mutagenesis approaches were used to demonstrate the stabilization of an aquaporin structure with the formation of disulfide bridges involving a conserved cysteine residue. Mutation at the cysteine position was found to increase water permeability as a result of the relaxation at the selectivity filter induced by the mutation.

## Tools and approaches for aquaporin study

Numerous tools and approaches have been developed over the last decade to study the aquaporin transport system. Notably, tools like the Xenopus oocyte system, yeast assays, excised membranes, and proteoliposomes assisted with flow cytometry have been developed to determine solute specificity and transport kinetics (Ampah-Korsah et al.; Deshmukh et al.). As stated previously, protein–protein and lipid–protein interactions play an important role in AQP regulation, and to study these aspects, recent advancements in X-ray crystallography, electron and confocal microscopy, the yeast two-hybrid system, co-precipitation assays, and immunodetection have offered improved opportunities. Review articles by Martínez-Ballesta and Carvajal, and Deshmukh et al. provide detailed examples of the implementation of new approaches for the study of AQPs.

## Genome-wide distribution, evolution and expression of aquaporins in plants

Facilitated access to annotated plant genome sequences and large transcriptomic and proteomic data has accelerated the omics scale of AQP research (Deokar and Tar'an; Rodrigues et al.; Zou et al.). Genome-wide identification and characterization of AQPs in many plant species have highlighted several interesting facts about these proteins. For instance, XIPs are missing from the entire monocots as well as from some dicots including the Brassicaceae members (Sonah et al., 2017). Similarly, NIP2s (NIP-III), including the ones recognized as Si transporters, are entirely absent in the Brassicaceae members, a finding that correlates well with the inability of these plants to uptake Si. In primitive plant species, two additional subfamilies, GlpF-like intrinsic protein (GIP) and hybrid intrinsic protein (HIP), have been observed (Deshmukh et al., 2015). The reason behind the loss of HIPs and GIPs in higher plants remains unexplained. Similarly, in spite of numerous recent genome-wide studies of AQPs, their distribution and evolution among plants are still not fully understood.

Expression profiling is a powerful approach to understand the functionality and regulation of AQPs. Recent advancements in sequencing technology have made transcriptome profiling more affordable and efficient, which has led to large amounts of publically available transcriptomic data (www.ncbi.nlm.nih.gov/sra). Using such resources, Deokar and Tar'an, Zou et al., and Song et al. have evaluated the expression of AQPs across different tissues and conditions. Using similar approaches, Shivaraj et al. (2017) observed seed specific expression of TIP3 genes in rice, Arabidopsis, soybean, and flax. As more expression studies across a wider range of plant species become available, a more precise understanding of the specific roles of AQPs should emerge.

## Role of AQPs in stress tolerance

Aquaporins are prime candidates for deciphering the mechanisms of abiotic stress tolerance in plants. Indeed, hundreds of reports link AQP expression with drought tolerance in plants. Heterologous expression of AQPs has been used to verify their functions under stress conditions. Alavilli et al. overexpressed HvPIP2;5, an AQP from *Hordeum vulgare*, in yeast and Arabidopsis, to investigate its role under high salinity and osmotic stress. In both systems, HvPIP2;5 was found to enhance tolerance to both forms of stress. The increased tolerance induced by HvPIP2;5 is presumably associated with increased expression and activities of reactive oxygen species scavenging enzymes such as catalase, superoxide dismutase, glutathione reductase, and ascorbate peroxidase. Similarly, Ding et al. also observed a correlation between ABA (stress related hormone) biosynthesis and expression of AQPs. Kadam et al. observed differential expression of AQPs among susceptible and tolerant genotypes under waterlogging condition. The expression profiling along with the haplotypic diversity for AQPs reported by Kadam et al. will be helpful for the development of waterlogging stress tolerance cultivars in sorghum

Unlike abiotic stress, very few studies have addressed the role of AQPs in plants during a biotic stress such as one caused by a pathogen. Along those lines, Deokar and Tar'an performed expression profiling of AQPs during fusarium wilt progression in chickpea. So far, most studies support a role for AQPs under stress conditions, but mechanistic hypotheses are still lacking to explain how their expression influences host-pathogen interactions.

## Outlook

Twenty-five years have elapsed since the seminal discovery of AQPs by Peter Agre's team in 1992 (Preston et al., 1992). Over that time period, significant progress was achieved in understanding the AQP transport system in numerous living organisms. Plants in particular have been the topic of intense research leading to major breakthroughs. Nevertheless, the study of AQPs in plants has proven particularly challenging compared to animals because of their higher number of homologs and more complex mechanisms of regulation. To comprehend the complex AQP system in plants, it will be fundamental to acquire a better knowledge of the co-regulation of AQPs, their interactions with other biomolecules and the influence of environmental conditions on their functionality and regulation. Similarly, knowledge of intracellular trafficking is important to understand AQP regulation, especially under adverse conditions. Studies addressing AQP intracellular trafficking have been greatly facilitated with the development of dynamic imaging techniques (Hachez et al., 2013; Luu and Maurel, 2013; Ueda et al., 2016). Nevertheless, more intense efforts are required to decipher how AQP intracellular trafficking co-ordinates a plant's response. In addition, emphasis should be placed on the structural components and their role on AQP permeability. Apart from water, very little is known about how other solutes are transported through AQP channels. To answer these questions, bioinformatics, sequence comparisons and high-resolution protein structure analyses are expected to contribute significantly to advance our understanding of plant AQPs.

## Author contributions

RD, HN, and RB have co-edited the topic and co-prepared the editorial.

### Conflict of interest statement

The authors declare that the research was conducted in the absence of any commercial or financial relationships that could be construed as a potential conflict of interest.
